# One-Step Hot Microembossing for Fabrication of Paper-Based Microfluidic Chips in 10 Seconds

**DOI:** 10.3390/polym12112493

**Published:** 2020-10-27

**Authors:** Yi-Je Juang, Yu Wang, Shu-Kai Hsu

**Affiliations:** 1 Department of Chemical Engineering, National Cheng Kung University, No.1 University Road, Tainan 70101, Taiwan; eva7773369@gmail.com (Y.W.); rfpjhnmf@gmail.com (S.-K.H.); 2 Center for Micro/nano Science and Technology, National Cheng Kung University, No.1 University Road, Tainan 70101, Taiwan; 3 Research Center for Energy Technology and Strategy, National Cheng Kung University, No.1 University Road, Tainan 70101, Taiwan

**Keywords:** microfluidics, filter paper, microembossing, wax, glucose detection

## Abstract

In recent years, microfluidic paper-based analytical devices (µPADs) have been developed because they are simple, inexpensive and power-free for low-cost chemical, biological and environmental detection. Moreover, paper is lightweight; easy to stack, store and transport; biodegradable; biocompatible; good for colorimetric tests; flammable for easy disposal of used paper-based diagnostic devices by incineration; and can be chemically modified. Different methods have been demonstrated to fabricate µPADs such as solid wax printing, craft cutting, photolithography, etc. In this study, one-step hot microembossing was proposed and demonstrated to fabricate µPADs. The processing parameters like embossing temperature, pressure and time were systematically investigated. It was found that, at 55 °C embossing temperature, the embossing pressure ranging from 10 to 14 MPa could be applied and the embossing time was only 5 s. This led to the overall processing time for fabrication of µPADs within 10 s. Glucose detection was conducted using the µPADs as fabricated, and a linear relationship was obtained between 5 and 50 mM.

## 1. Introduction

Since the 1980 s, microfluidics has emerged as an attractive research subject and been widely studied and explored for potential applications such as biomedical applications [1,2], drug screening [3,4], point of care applications [5,6,7], and environmental monitoring [8,9,10], for chemical and biological detection [11,12], etc. This is because of their competitive advantages over conventional analytical methods like fast response, high throughput, low reagent consumption, reduced waste product, high sensitivity and great portability. Among the commonly used materials for microfluidic devices such as silicon, glass, polymer and elastomer [13,14], paper is considered an attractive alternative because of its low cost and ubiquity [15]. In addition, the hydrophilicity and porous nature of paper means it possesses high surface-to-volume ratio, being capable of power-free fluid transport via capillary and suitable for reagent storage [16] and colorimetric tests. Moreover, paper is biodegradable, biocompatible and flammable for easy disposal by incineration [17]. With the potential utility in point-of-care testing (POCT) and onsite analyses, microfluidic paper-based analytical devices (µPADs) have become a promising alternative to traditional laboratory experiments [18,19].

Numerous methods have been demonstrated to fabricate µPADs such as photolithographic technique, ink jet printing, cutting/shaping and wax printing [16,20]. For the photolithographic technique, filter paper is patterned by photoresist, which forms solid barriers as the channel wall that can achieve high resolution and small dimension. However, the cost of fabrication is relatively high and there are issues related to crack of chip and channel contamination [21]. Ink jet printing creates barriers by printing special ink, but it may not be suitable for mass fabrication due to the throughput [22]. Cutting/shaping is simple and easy; however, the devices suffer low mechanical strength and warpage [16]. Moreover, the process becomes lengthy when the design of the microfluidics is complicated. Currently, wax printing is widely used to construct µPADs owing to its low cost, simplicity and relative rapidness and the total process duration can be reduced to 5–10 min. [23,24]. Studies were conducted to investigate spreading of wax and resolution of the device, which was found to be dependent on the applied pressure, temperature and types of the filter paper [25]. Despite the advantages, energy consumption is a concern as the temperature of heating wax in most of the cases is above 100 °C and the heating time is approximately 3–5 min. In addition, the overall processing time also depends on complexity of the microfluidic design. Other techniques involved in utilization of the mold that are potentially more scalable, like stamping, calendaring and microembossing, have been developed to fabricate µPADs [26,27,28,29,30]. For this category of methods, construction of the microchannels is to use either a preheated, patterned metal stamp to press the paraffin-filled paper against the clean filter paper [26] or a heated stamp to press the photolithographically patterned parafilm against the filter paper [27]. Another way is to create the trenches on the filter paper. For example, Oyola-Reynoso et al. used a ball point pen filled with the customized ink to engrave the channel on the filter paper. During the process, the customized ink was left along the channel and subsequently used to hydrophobize the channel [28]. Thou et al. demonstrated the utilization of two molds with complementary shapes to deform the filter paper, and an open channel was obtained, which was then treated with silanization and sealed with a tape to form the closed channel [29]. Without using wax (or other hydrophobic materials), simply applying high pressure to emboss the nonwoven polypropylene sheet can lead to the formation of a barrier [30]. This is because, at the embossed area, the polypropylene fibers were physically bonded together such that the gaps were closed and the embossed area acted as the barrier to prevent the flow of fluids from leakage. In recent years, a true microembossing method was developed by our research group to fabricate the µPADs [31]. With this method, the filter paper was subjected to a compression force exerted by a metallic female mold, and the embossed filter paper with protruded microstructures was obtained. After applying the wax to the backside of the embossed filter paper and heating it, the wax diffused into the filter paper for a certain time to form the barrier without completely blocking the protruded microstructures, which were then used as the microchannels. The processing conditions were as follows: 75 °C embossing temperature, 5 s embossing time, and 12 MPa embossing pressure. The overall processing time for construction of the µPADs was approximately 1 min.

To extend our work with the goal to further reduce the processing time and the energy consumption, we proposed to combine the microembossing and heating processes, i.e., one-step hot microembossing to fabricate µPADs. Different processing conditions such as embossing temperature, pressure and time were discussed. In addition, the glucose detection was performed using the 00B5PADs as fabricated.

## 2. Materials and Methods

### 2.1. Fabrication of Paper-Based Microfluidic Chips

**Figure 1:**
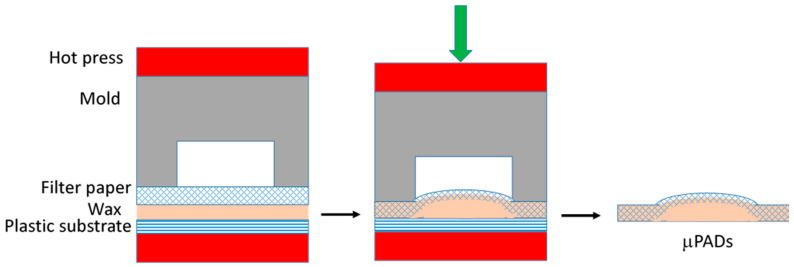
Schematics of one-step hot microembossing process (not in scale).

**Figure 2:**
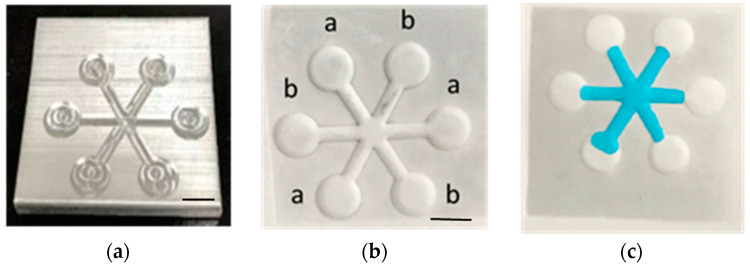
(**a**) The metalic mold for the microembossing process. (**b**) The embossed filter apper. (**c**) The channels with ink wicking through. Scale bar: 4 mm.

Figure 1 shows the schematics of one-step hot microembossing. A metallic mold was milled by the computer numerical controlled (CNC) machine (Roland EGX-400, Twinsoft Co., New Taipei City, Taiwan). The DLC-coating (diamond-like-carbon coating) endmills with different end diameters were used. A pattern with six channels connected by a central reservoir was constructed, and each channel has a reservoir at the end as shown in Figure 2a. The length of the channel, radius of the central reservoir, and that of the end reservoir were 12, 2, and 2 mm, respectively. The width of the channel was between 2 and 4 mm. The depth of the channel was 0.6 mm. Prior to fabrication, a plastic substrate was dip-coated into the melted paraffin wax (Sigma-Aldrich, St. Louis, MO, USA), followed by cooling in the ambient. The filter paper (Whatman #1, 3, and 4; GE Healthcare, Chicago, IL, USA) was then placed on top of the plastic substrate and in contact with wax, followed by placing the mold on top of the filter paper. The assembly was then placed inside the embossing machine (QC-601T, Cometech, Taichung, Taiwan) under pressure. The operating temperature was set between 50 and 65 °C, the embossing pressure was applied between 10 and 16 MPa, and the embossing time ranged from 5 to 10 s. After embossing, the embossed filter paper with the designed pattern was separated from the mold and the plastic substrate, which was ready for use. For flow visualization and characterization, the µPAD was placed under the microscope (SMZ-745T, Nikon, Tokyo, Japan) and the ink solution was dispensed in the central reservoir. The flow was recorded by the computer software.

### 2.2. Glucose Detection

The fabricated paper-based microfluidic chips were used to perform glucose detection through enzymatic oxidation of iodide to iodine. Preparation of the glucose solution can be found in the literature [31]. In brief, 1.5 µL of potassium iodide solution (0.6 M) was first dispensed at the detection zone. After drying under ambient condition, 1.5 µL of horseradish peroxidase-glucose oxidase enzyme mixture (1:5) was spotted at the detection zone. Then, 20 µL of the glucose solution with concentrations ranging from 1 to 50 mM (in pH 7.4 buffer) was then dispensed at the loading zone (i.e., the central reservoir). The resultant colour changes were observed under the microscope. The chip was then placed under the ambient condition for 15 min prior to taking a photo image and scanning.

Quantification of the color response was carried out by using a commercially available scanner (HP, Photosmart C4580) to capture the images of the detection zone, which were deconvoluted into red (R), green (G) and blue (B) components by computer software [31]. The intensity was taken as quantification of the color image.

## 3. Results and Discussion

### Fabrication of Paper-Based Microfluidic Chips by One-Step Hot Microembossing

**Figure 3:**
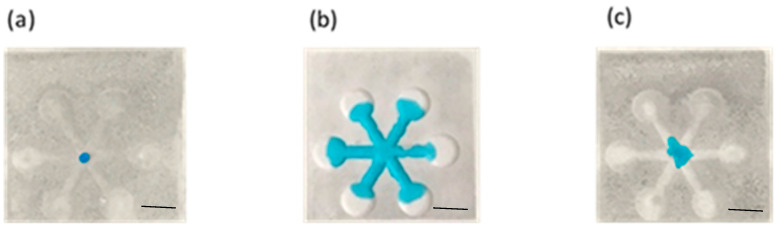
Dispensing ink solution on the embossed filter paper. (**a**) Whatman #1. (**b**) Whatman #3. (**c**) Whatman #4. Scale bar: 4 mm.

**Figure 4:**
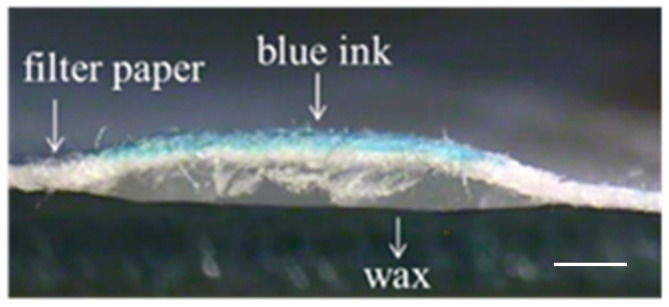
The cross-sectional view of the embossed filter paper with ink solution wicking through the channel. Scale bar: 1 mm.

Figure 2b shows the embossed, paper-based microfluidic chip. It can be seen that the filter paper in contact with the mold was compressed downward and a protruded structure was formed at the location where the filter paper was not in contact with the mold. The embossing temperature and pressure were set at 55 °C and 12 MPa, respectively, and the processing time was approximately 5 s. After dispensing the ink solution at the center reservoir, it wicks through the channel without leakage as shown in Figure 2c. The smallest channel width fabricated by our proposed method was approximately 1 mm; however, successful and consistent test results were obtained when it was 2 mm and larger. The method was also applied to different filter papers, and it was found that there is channel blockage when using both Whatman #1 and #4, as shown in Figure 3. For Whatman #1 (thickness: 180 µm, pore size: 11 µm) and Whatman #4 (thickness: 205 µm, pore size: 25 µm), the thickness is smaller but the pore size is larger than those of Whatman #3 (thickness: 390 µm, pore size: 6 µm). This might allow the melted wax to fill the filter paper completely within a short period of time. Although further decreasing the embossing time to less than 5 s may prevent the melted wax from completely filling the filter paper, the process is simply impractical in terms of complexity in system control and quality assurance. Therefore, the Whatman #3 was used throughout this study. To better understand how the channel was formed, the cross-sectional area of the ink-filled channel was examined. It can be seen that the filter paper was bulged at the unembossed region where there was wax beneath the filter paper, as shown in Figure 4. The blue ink was confined at the top of the filter paper, which was the channel. It is shown that the wax at the embossed region is squeezed under compression, which moves laterally to the unembossed region and causes the filter paper to bulge. Meanwhile, the wax is heated and diffuses into the filter paper. Therefore, for proper embossing time, only top of the filter paper at the unembossed region does not get completely filled with wax.

**Figure 5:**
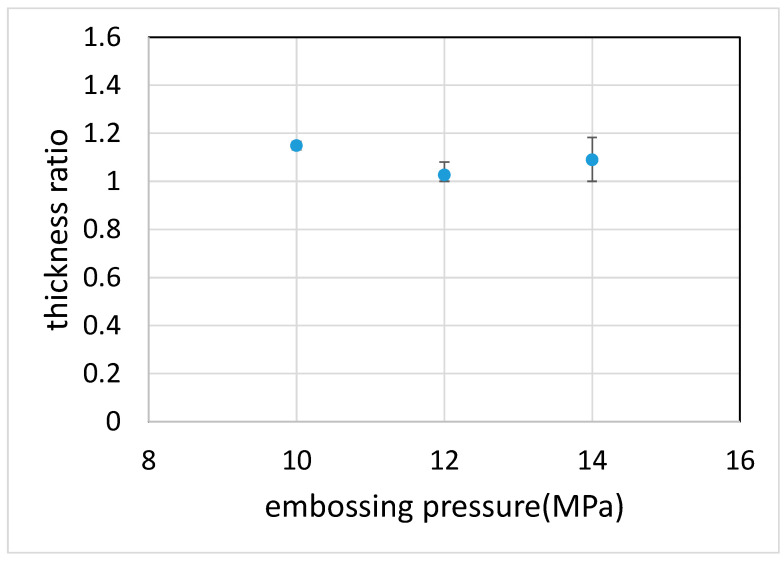
The thickness ratio at different embossing pressure. The thickness ratio was defined as the thickness of the filter paper in the unembossed region divided by that in the embossed region.

Figure 5 shows the effect of compression pressure on the thickness of the filter paper. The thickness ratio was defined as the thickness of the filter paper at the unembossed region divided by that at the embossed region. It can be seen that the thickness of the filter paper at the unembossed region was approximately 10% thicker (approximately 25 µm) than that at the embossed region. This indicates that there may need to be more wax to completely fill the filter paper at the unembossed region. Moreover, since the thickness of the wax beneath the filter paper in the embossed region is smaller than that in the unembossed region, it reaches the melting temperature faster and thus diffuses into the filter paper earlier than the later. These may explain why, for the proper embossing time, the wax can form the barrier at the embossed region without completely filling the filter paper in the unembossed region.

**Figure 6:**
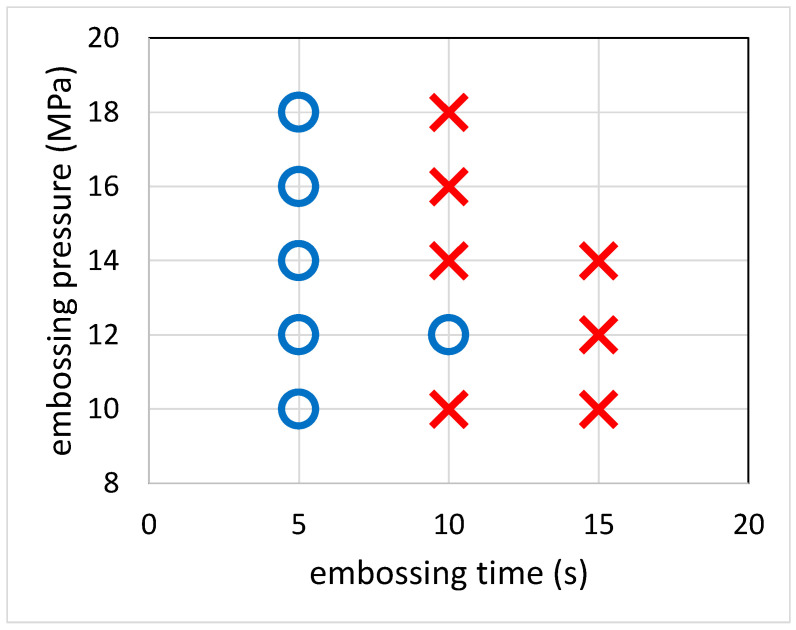
Effect of embossing pressure and embossing time on the process. The symbol “circle” refers to successful channel formation while the symbol “cross” refers to channel blockage.

Based on the previous argument, it is clear that there is an interplay between several processing parameters such as embossing temperature, embossing time and embossing pressure. To determine the embossing temperature, the DSC analysis of the wax was carried out and the melting temperature was found to be around 60 °C. Starting with 55 °C embossing temperature, it was found that the paper-based microfluidic chips can be fabricated in 5 s under various embossing pressures, as shown in Figure 6. Moreover, there was no leakage observed as the ink solution wicked through the channels. As the embossing time increased to 10 s, channel blockage was observed. Note that, although the paper-based microfluidic chips were successfully constructed at the embossing pressure 12 MPa for 10 s embossing time, either channel blockage was observed for some channels or the successful rate was not high. It can be rationalized that increasing the embossing time allows the wax to diffuse further into the filter paper and be able to completely fill it. This became conspicuous as we further increased the embossing time, and no successful paper-based microfluidic chips were obtained. At 60 °C embossing temperature, it was found that 5 s was sufficient enough for the wax to diffuse into and fill the filter paper completely, and channel blockage was observed at all processing conditions. On the other hand, at 50 °C embossing temperature, channel leakage was observed and even the embossing time was increased up to 15 s. This indicates that the wax either did not melt or melted slightly such that the barriers did not properly form.

**Figure 7:**
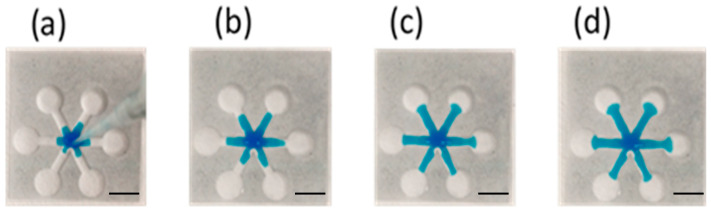
The sequential images of ink wicking through the channels at (**a**) 1, (**b**), 4, (**c**) 8 and (**d**) 12 s. Scale bar: 4 mm.

**Figure 8:**
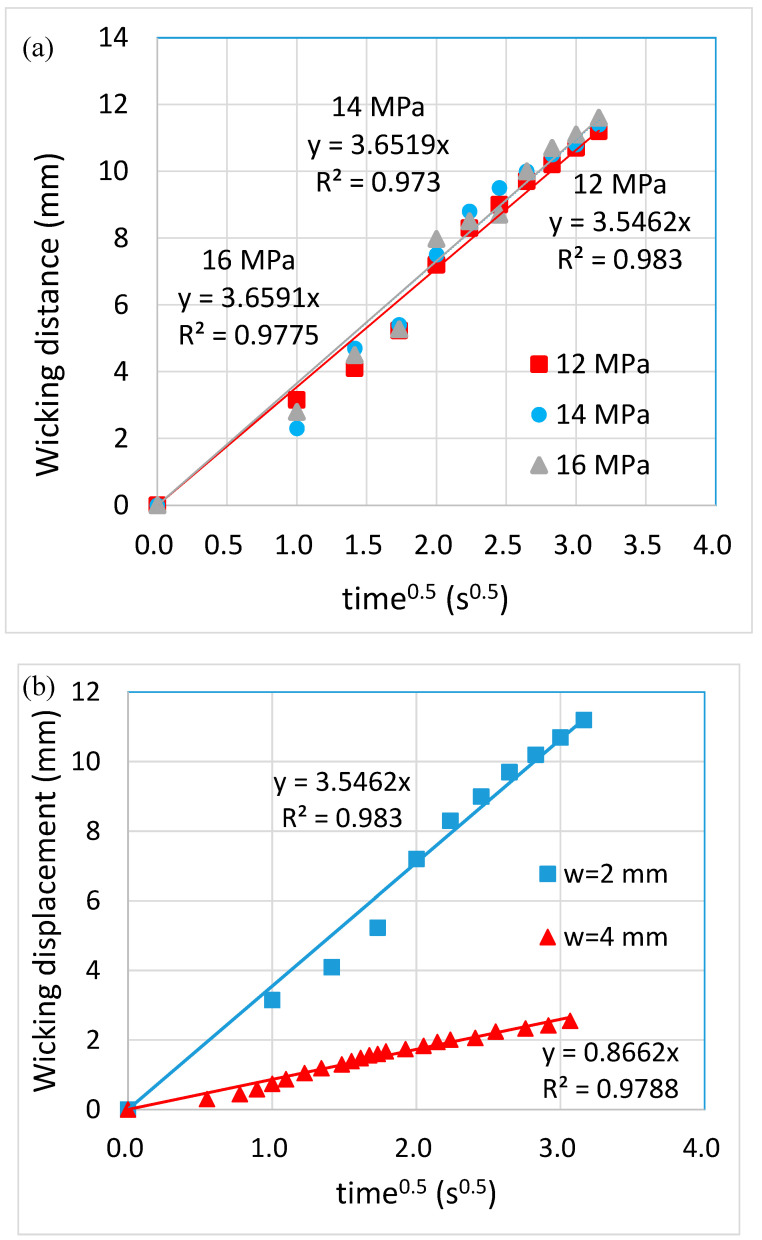
The flow behavior of solution wicking through (**a**) 2-mm wide channels fabricated using different embossing pressures and (**b**) channels with different widths fabricated using 12 MPa embossing pressure.

**Figure 9:**
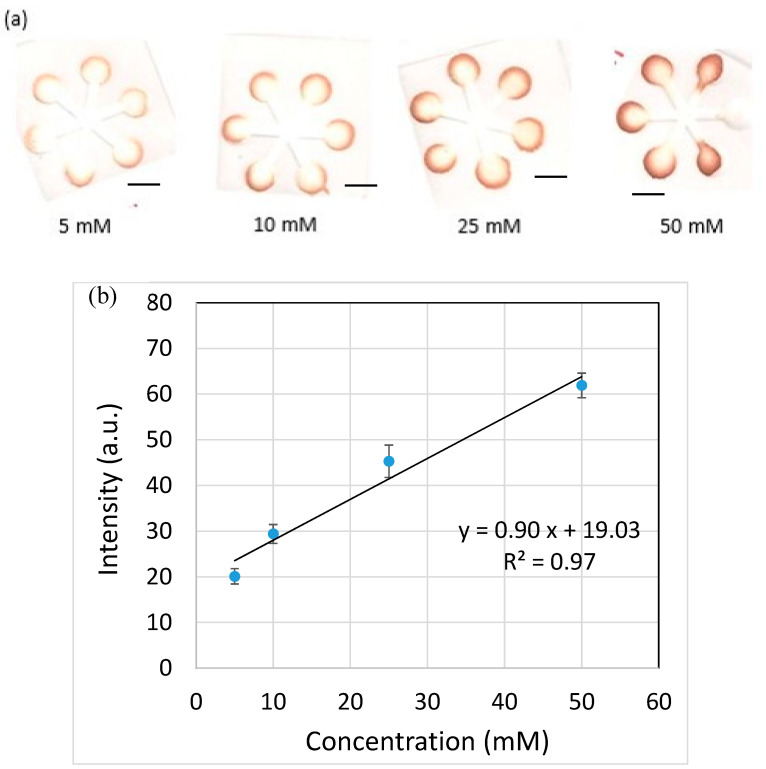
(**a**) The color images of the paper-based microfluidic chips for glucose detection. (**b**) The relationship between color intensity and detected glucose concentration. Scale bar: 4 mm.

Figure 7 shows the sequential images of the flow behavior. It can be seen that there was neither blockage nor leakage when the ink wicked through the channels. Moreover, the ink reached the reservoirs at approximately the same time, indicating the consistency among the channels. The flow behavior can be characterized using Washburn’s equation, as shown in the following:

(1) $$L=S\sqrt{t},\;S=\sqrt{\frac{\gamma Dcos\theta }{4\mu }}$$

where L is the wicking distance, *γ* is the liquid-vapor interfacial tension, *D* is the average pore diameter, *θ* is the contact angle for the three-phase system, *μ* is the liquid viscosity and t is the time for liquid to wick through the distance L. It can be seen that not only there existed a linear relationship between L and $\sqrt{t}$, as shown in Figure 8a, but the linear relationships almost overlapped with each other for different embossing pressures as well. This supports our previous findings regarding the thickness of the filter paper, that is, the embossing pressure did not alter the thickness of the filter paper significantly. On the other hand, the flow behavior was substantially influenced by the channel width, as shown in Figure 8b. It is consistent with the results from the literature that the larger the channel width, the slower the flow rate. This is because more liquid needs to fill the pores as the channel width increases, thus reducing the flow rate of the liquid front. The glucose detection was also carried out using the paper-based microfluidic chips as fabricated, as shown in Figure 9a. The brown color became darker as the glucose concentration increased. The intensity was measured individually, and the averaged value was taken. A linear relationship was obtained at the detection range from 5 to 50 mM, as shown in Figure 9b.

Table 1 shows the comparison between commonly used techniques and our proposed method. It can be seen that, although a mold is required and the equipment cost is relatively higher, substantial reduction of overall processing time is the major advantage, which will be much more appreciated if the design of microfluidics becomes complicated. Other merits such as energy saving (i.e., lower temperature applied) and easy maintenance render this method potentially scalable.

## 4. Conclusions

In this paper, we proposed and demonstrated a novel approach for rapid fabrication of paper-based microfluidic chips. By combining the embossing and heating processes together, it allows diffusion of the melted wax into the filter paper to simultaneously create the channel and channel barrier. This leads to the overall processing time within 10 s. The thickness of the filter paper did not alter very much at different embossing pressures. In addition, the channels constructed by using different embossing pressures showed the similar flow behavior. The flow behavior was, however, influenced by the channel width. That is, the larger the channel width, the slower the flow rate. The glucose detection was carried out using the paper-based microfluidic chips as fabricated, and a linear relationship was obtained between 5 and 50 mM glucose concentration.

## Figures and Tables

**Table 1:** Comparison between commonly used techniques and proposed method.

	Processing Time (s)	Heating Time (s)	Heating Temperature (°C)	Mold	Equipment Cost ($)	Potential for Scalability
Solid wax printing	Depends on μPAD design	30–600 [32]	100–175 [32]	No	~500	Medium
Craft cutting	Depends on μPAD design	No	No	No	~250	Medium
One-step hot microembossing	~10	~5	~55	Yes	~3000	High
